# Anti-Allergic, Anti-Inflammatory, and Anti-Hyperglycemic Activity of *Chasmanthe aethiopica* Leaf Extract and Its Profiling Using LC/MS and GLC/MS

**DOI:** 10.3390/plants10061118

**Published:** 2021-05-31

**Authors:** Iriny M. Ayoub, Michal Korinek, Mohamed El-Shazly, Bernhard Wetterauer, Hesham A. El-Beshbishy, Tsong-Long Hwang, Bing-Hung Chen, Fang-Rong Chang, Michael Wink, Abdel Nasser B. Singab, Fadia S. Youssef

**Affiliations:** 1 Department of Pharmacognosy, Faculty of Pharmacy, Ain Shams University, Abbassia, Cairo 11566, Egypt; irinyayoub@pharma.asu.edu.eg (I.M.A.); mohamed.elshazly@pharma.asu.edu.eg (M.E.-S.); fadiayoussef@pharma.asu.edu.eg (F.S.Y.); 2 Graduate Institute of Natural Products, College of Pharmacy, Kaohsiung Medical University, Kaohsiung 80708, Taiwan; mickorinek@hotmail.com; 3 Department of Biotechnology, College of Life Science, Kaohsiung Medical University, Kaohsiung 80708, Taiwan; bhchen@kmu.edu.tw; 4 Graduate Institute of Natural Products, College of Medicine, Chang Gung University, Taoyuan 33302, Taiwan; htl@mail.cgu.edu.tw; 5 Research Center for Chinese Herbal Medicine, Research Center for Food and Cosmetic Safety, and Graduate Institute of Health Industry Technology, College of Human Ecology, Chang Gung University of Science and Technology, Taoyuan 33302, Taiwan; 6 Department of Pharmaceutical Biology, Faculty of Pharmacy and Biotechnology, German University in Cairo, Cairo 11835, Egypt; 7 Institute of Pharmacy and Molecular Biotechnology, Heidelberg University, INF 364, D-69120 Heidelberg, Germany; bernhard.wetterauer@uni-heidelberg.de; 8 Medical Laboratory Sciences Department, Fakeeh College for Medical Sciences, Jeddah 21461, Saudi Arabia; hesham_elbeshbishy@hotmail.com; 9 Biochemistry and Molecular Biology Department, Faculty of Pharmacy, Al-Azhar University, Nasr City, Cairo 11231, Egypt; 10 Department of Anesthesiology, Chang Gung Memorial Hospital, Taoyuan 33302, Taiwan; 11 Drug Development and Value Creation Research Center, Kaohsiung Medical University, Kaohsiung 80708, Taiwan; 12 Department of Medical Research, Kaohsiung Medical University Hospital, Kaohsiung Medical University, Kaohsiung 80708, Taiwan; 13 Department of Marine Biotechnology and Resources, National Sun Yat-sen University, Kaohsiung 80424, Taiwan; 14 Center for Drug Discovery Research and Development, Faculty of Pharmacy, Ain Shams University, Abbassia, Cairo 11566, Egypt

**Keywords:** anti-allergic, antihyperglycemic, *Chasmanthe aethiopica*, anti-inflammatory, GC/MS, LC/MS

## Abstract

This study aims to comprehensively explore the phytoconstituents as well as investigate the different biological activities of *Chasmanthe aethiopica* (Iridaceae) for the first time. Metabolic profiling of the leaf methanol extract of *C. aethiopica* (CAL) was carried out using HPLC-PDA-ESI-MS/MS. Twenty-nine compounds were annotated belonging to various phytochemical classes including organic acids, cinnamic acid derivatives, flavonoids, isoflavonoids, and fatty acids. Myricetin-3-*O*-rhamnoside was the major compound identified. GLC/MS analysis of the *n*-hexane fraction (CAL-A) resulted in the identification of 45 compounds with palmitic acid (16.08%) and methyl hexadecanoic acid ester (11.91%) representing the major constituents. CAL-A exhibited a potent anti-allergic activity as evidenced by its potent inhibition of *β*-hexosaminidase release triggered by A23187 and IgE by 72.7% and 48.7%, respectively. Results were comparable to that of dexamethasone (10 nM) in the A23187 degranulation assay showing 80.7% inhibition for *β*-hexosaminidase release. Both the *n*-hexane (CAL-A) and dichloromethane (CAL-B) fractions exhibited potent anti-inflammatory activity manifested by the significant inhibition of superoxide anion generation and prohibition of elastase release. CAL showed anti-hyperglycemic activity in vivo using streptozotocin-induced diabetic rat model by reducing fasting blood glucose levels (FBG) by 53.44% as compared with STZ-treated rats along with a substantial increase in serum insulin by 22.22%. Molecular modeling studies indicated that dicaffeoylquinic acid showed the highest fitting with free binding energies (∆G) of −47.24 and −60.50 Kcal/mol for human *α*-amylase and *α*-glucosidase, respectively confirming its anti-hyperglycemic activity. Thus, *C. aethiopica* leaf extract could serve as an effective antioxidant natural remedy combating inflammation, allergy, and hyperglycemia.

## 1. Introduction

Oxidative stress can be defined as the disturbance in the balance between antioxidants and free radicals within the body that leads to cell and tissue destruction. During the normal process of metabolism, the body releases free radicals with the concomitant production of natural antioxidants to antagonize and neutralize these free radicals. However, a multitude of factors results in the massive production of free radicals that exaggerates oxidative stress including the unhealthy diet and lifestyle, environmental factors such as exposure to radiation and pollution as well as the natural body immune response that temporarily contributes to oxidative stress [1]. Oxidative stress can provoke an inflammation that consequently releases excess free radicals that in turn promote more oxidative stress resulting in a vicious cycle. Chronic exposure to inflammation because of oxidative stress can elicit various human ailments such as diabetes mellitus, allergy, and cardiovascular disease in addition to cancer, and neurodegenerative disorders [2]. ROS can oxidize guanosine in DNA to 8-oxoguanosine which can lead to mutations and in consequence the genetic diseases such as cancer.

Diabetes mellitus has been recently considered the third leading cause of death following cancer and cardiovascular disorders with high prevalence all over the globe [3]. It adversely affects human health causing serious harmful changes in all parts of the body particularly the nerves and blood vessels [4]. Allergy is another global health threat that is also triggered by oxidative stress. It can threaten life in cases of severe asthma and anaphylaxis and can also interfere with the quality of life in cases of chronic allergic conditions exemplified by allergic rhinitis and eczema [5]. Despite the presence of numerous synthetic agents that could alleviate inflammation, relieve allergy, or manipulate hyperglycemia, drugs of natural origin always constitute the main key player in curing various human ailments due to their acceptable prices and safety in comparison with synthetic agents [6].

*Chasmanthe aethiopica* (Iridaceae) (syn. *Antholyza aethiopica* L.) is native to South Africa and is characterized by being a deciduous, bulbous plant that grows up to a height of 0.6 m with pale green lanceolate leaves [7]. Although members of family Iridaceae are popular by the presence of a wide array of compounds represented by flavonoids, isoflavonoids, xanthones, and quinones to which numerous biological activities including antioxidant, anti-inflammatory, antidiabetic, and phytoestrogenic effects are attributed [8,9], nothing was found in the literature regarding the phytochemical profile or the biological potential of *C. aethiopica.*

This study aims to comprehensively study the phytoconstituents as well as biologically investigate the different activities of the plant for the first time. Herein, we evaluated the anti-allergic and anti-inflammatory activity of the 80% methanol leaf extract of *C. aethiopica* (CAL) as well as its successive fractions in vitro. Additionally, the antihyperglycemic potential of CAL was assessed using streptozotocin-induced diabetes in a rat model. Moreover, metabolic profiling of CAL and its bioactive fractions was performed using LC/MS (Liquid Chromatography coupled with Mass Spectrometry) and GLC/MS (Gas–Liquid Chromatography coupled with Mass Spectrometry). To understand the reasons behind the antihyperglycemic activity, molecular modeling experiments were performed on the identified compounds from the bioactive fractions using human *α*-glucosidase and *α*-amylase, as crucial enzymes entangled in the occurrence and dissemination of diabetes.

## 2. Results and Discussion

### 2.1. Gas–Liquid Chromatography Coupled with Mass Spectroscopy (GLC-MS) Analysis

**Figure 1:**
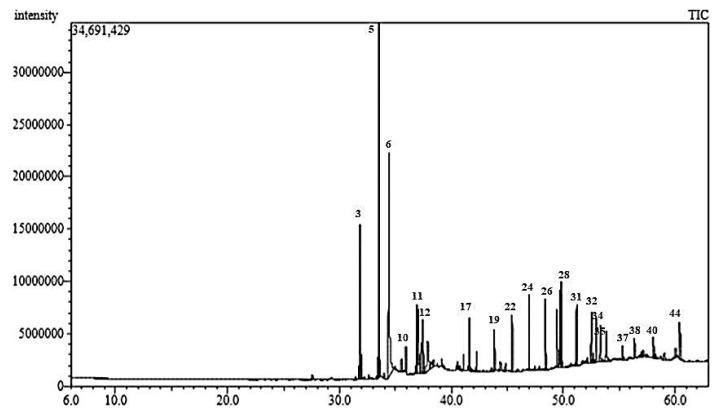
GLC-chromatogram of *C. aethiopica* L. *n*-hexane fraction (CAL-A) on Rtx-5MS column.

Characterization of the *n*-hexane fraction (CAL-A) of the leaf methanol extract (CAL) of *C. aethiopica* using GLC analysis (Figure 1) resulted in the tentative identification of 45 compounds belonging to sterols and fatty acids accounting for 91.62% of the total *n*-hexane fraction constituents as illustrated in Table 1. Palmitic acid (16.08 %) and methyl hexadecanoic acid ester (11.91%) represent the major constituents in the CAL-A fraction.

### 2.2. HPLC-PDA-ESI-MS/MS Analysis for Characterization of C. aethiopica Methanol Extract

**Figure 2:**
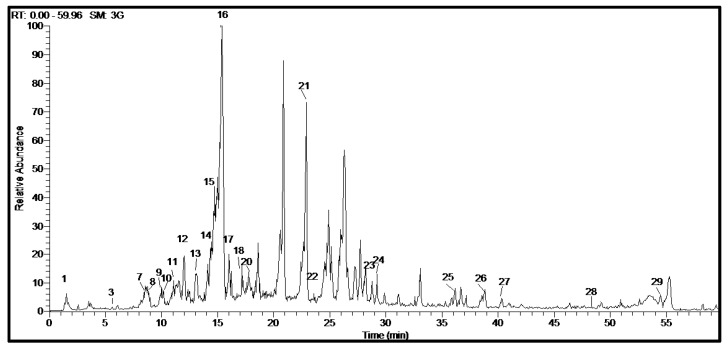
HPLC-ESI-MS base peak chromatogram of *C. aethiopica* leaf total methanol extract in the negative ion mode.

**Figure 3:**
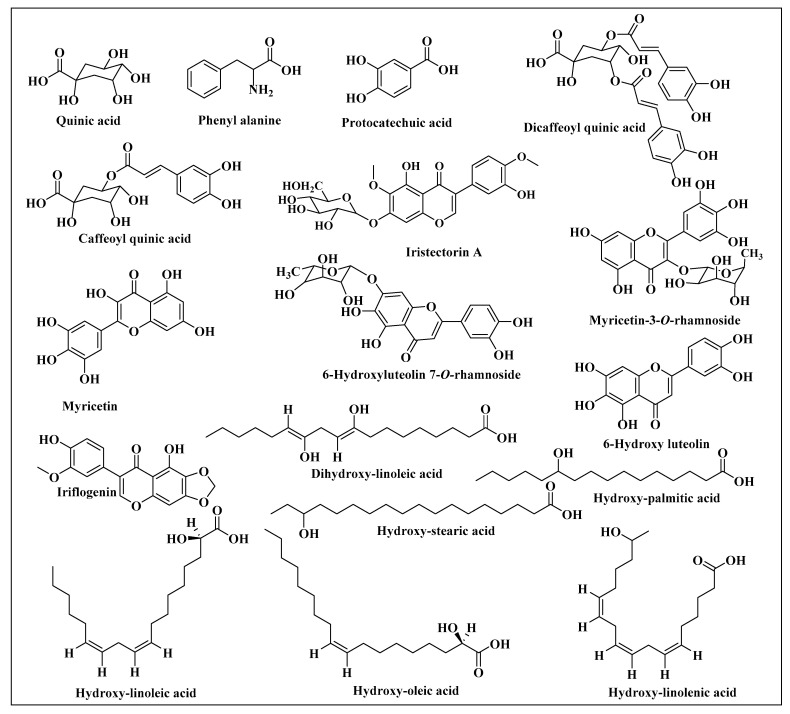
Major compounds identified in *C. aethiopica* leaf total methanol extract using HPLC-ESI-MS in the negative ion mode.

Metabolite profiling of *C. aethiopica* total leaf extract (CAL) was carried out using HPLC-PDA-ESI-MS/MS analysis. A total of 29 chromatographic peaks were annotated using simultaneously acquired HPLC–PDA, and HPLC–MS base peak chromatograms (Figure 2). Tentative metabolite assignments were achieved by comparison of mass and UV spectral data of the detected compounds in both negative and positive ionization modes with reported data alongside online public databases. The identified secondary metabolites are listed in Table 2 along with their spectroscopic data. Compounds were illustrated in Figure 3. Several classes of compounds were identified in *C. aethiopica* total leaf extract including organic acids, cinnamic acid derivatives, flavonoids, isoflavonoids, and fatty acids. Flavonoids represented the most abundant class of metabolites detected in CAL. This is the first comprehensive metabolites profiling of genus *Chasmanthe* using HPLC-ESI-MS.

#### 2.2.1. Flavonoids

Flavonols, mainly myricetin, in addition to several distinct flavones, markedly tricin and 6-hydroxyluteolin derivatives, were reported in Ixieae as important chemotaxonomic markers [10,11]. In the current study, tricin-*O*-rhamnoside (**12**), myricetin-3-*O*-rhamnoside (**15**), myricetin (**16**), 6-hydroxyluteolin-*O*-rhamnoside (**18**), and 6-hydroxyluteolin (**19**) were identified in *C. aethiopica* methanol extract. A mass loss of 308 amu suggested a loss of 6-rhamnosylhexose (rutinose) or a *p*-coumaroylhexose. However, acylation with *p*-coumaric acid results in a typical bathochromic shift in band I to λ_max_ of 310–316 nm in the ultraviolet/visible (UV–VIS) spectra of flavonols and a pseudo molecular ion that is 146 amu greater than the parent glycoside. Moreover, acylation of the sugar residue causes a subsequent increase in the retention time in a chromatographic analysis [12,13]. Myricetin-*O*-coumaroyl-hexoside (**9**, **11**) and myricetin-*p*-coumaroyl-rhamnosyl-hexoside (**20**) were identified herein, exhibiting *λ*_max_ of 310–316 nm and pseudomolecular ions [M − H]^−^ at *m/z* 625 and 771, respectively.

Several isoflavonoids were identified in *C. aethiopica* methanol extract including iristectorin A/B (**10**), irigenol-*C*-hexoside (**14**), hydroxy-iristectrigenin-*O*-hexoside (**17**), iriflogenin (**21**), dihydroxy-methoxy-6,7-methylenedioxy isoflavone (**22**). Mass spectrometry cannot generally distinguish isoflavones from flavones, where both classes follow a similar fragmentation pathway via retro Diels–Alder (RDA) fragmentation resulting in identical product ions for both classes of compounds [14]. However, conjugation between A- and B-rings is lacking in isoflavone structures. Thus, UV spectra of isoflavones could be distinguished from those of flavones by displaying a low-intensity band I absorption. Moreover, the increased A-ring oxygenation results in a bathochromic shift in band II from 249 nm in 7,4′-dihydroxyisoflavone to 261 nm in 5,7,4′-trihydroxyisoflavone and 270 nm in 5,6,7,4′-tetrahydroxyisoflavone. The spectra of isoflavones are not affected by changes in B-ring oxygenation patterns [14].

#### 2.2.2. Fatty Acids

Several fatty acids could be annotated in the second half of the base peak chromatogram in ESI negative mode of *C. aethiopica* methanol extract exhibiting intense [M − H]^−^ pseudo-molecular ions characteristic of fatty acids. Monohydroxy unsaturated fatty acids including hydroxy-octadecatrienoic acid (**25**), hydroxy-octadecadienoic acid (**26**), and hydroxy-octadecenoic acid (**27**) could be detected exhibiting quasi molecular ions [M − H]^−^ at *m/z* 293, 295, 297, respectively. A mass difference of 2 amu between the identified fatty acids indicates an additional double bond [15]. A dihydroxy polyunsaturated fatty acid was identified as dihydroxy-octadecadienoic acid (**24**) exhibiting [M − H]^−^ at *m/z* 311. Monohydroxy saturated fatty acids including hydroxy-hexadecanoic acid (**28**) and hydroxy-octadecanoic acid (**29**) were also assigned. Moreover, dihydroxy-23-oxo-12-oleanen-28-oic acid-*O*-pentosyl dihexoside (**23**) was identified in *C. aethiopica* methanol extract.

Miscellaneous compounds including quinic acid (**1**), phenylalanine (**2**), protocatechuic acid (**3**), dicaffeoyl quinic acid and its isomer (**4**–**5**), hydroxy-methoxy acetophenone-*O*-hexoside (**6**), caffeoyl quinic acid and its isomer (**7**–**8**) were also identified in *C. aethiopica* methanol extract.

### 2.3. In Vitro Evaluation of the Anti-Allergic Activity

*C. aethiopica* leaf total methanol extract (CAL), as well as its various fractions, were assessed for their anti-allergic potential using A23187- and antigen (IgE+DNP-BSA)-induced degranulation assays, and the results were illustrated in Table 3. The cytotoxic effect against rat basophilic leukemia (RBL-2H3) for all the samples was evaluated using MTT assay. All samples exhibited toxic effects to RBL-2H3 cells (viability below 80%) at concentration ≥ 10 μg/mL except for CAL-A which showed no cytotoxicity at the tested doses. Samples that were nontoxic were further examined for their anti-allergic effect. All the tested samples revealed weak inhibition percentage in both degranulation assays as illustrated in Table 3 except for CAL-A. It exhibited potent anti-allergic activity as evidenced by its pronounced inhibition percentage on *β*-hexosaminidase release triggered by A23187 and IgE by 72.7 and 48.7%, respectively, approaching that of dexamethasone especially in A23187 degranulation assay that revealed 80.7% inhibition for *β*-hexosaminidase release at 10 nM. This considerable anti-allergic activity comes following the anti-asthmatic and anti-allergic potential of previously studied Iridaceae plants such as *Crocus sativus* [34] and *Dietes bicolor* [35]. The hydroxy fatty acids and their esters, the predominant compounds in CAL-A, were previously reported to possess a significant anti-allergic activity as demonstrated in vitro and by ChemGPS-NP (chemical global positioning system for natural products) [36].

### 2.4. In Vitro Evaluation of Anti-Inflammatory Activity

The anti-inflammatory activity of *C. aethiopica* leaf total methanol extract and its fractions on superoxide anion generation and elastase release in FMLF/CB-induced human neutrophils was evaluated. Results displayed in Table 4 showed that both *n*-hexane (CAL-A) and dichloromethane (CAL-B) fractions revealed potent anti-inflammatory activity manifested by significant inhibition in the generation of superoxide anion and prohibition of elastase release. CLA-A inhibited the generation of superoxide anion and elastase release by 68.68 and 65.18%, respectively at 10 μg/mL showing IC_50_ equal to 5.32 and 5.8 μg/mL, respectively. CAL-B exhibited 58.31 and 68.08% inhibition of superoxide anion generation and elastase release, respectively, at 10 μg/mL exhibiting IC_50_ values of 7.04 and 5.73 μg/mL, respectively. Many Iridaceae plants showed potent anti-inflammatory and immunomodulating effects in previous studies [35]. Noteworthy to highlight that the cellular model of isolated human neutrophils was established to elucidate the anti-inflammatory effect in this study as neutrophils are considered the first line of defense versus microbial infections in addition to being the most abundant circulating leukocytes. Moreover, they greatly contribute to the damage of tissues during several autoimmune and inflammatory diseases [37]. Elastase is a serine protease released by activated human neutrophils and can break several biomarkers comprising elastin in addition to being considered as inflammatory disorders marker. Initiation of elastase release is triggered by formyl peptides such as fMLF in neutrophils; thus, efficient elastase inhibitors can act as a logic strategy to protect tissue from excessive damage caused by inflammatory mediators [38]. The superoxide radical plays a pivotal role in the neutrophil-medicated acute inflammatory response. Superoxide radicals are produced by neutrophils to help in killing invasive microbes where superoxide evolved from actively phagocytizing neutrophils that attracts additional neutrophils via reaction and activation of latent chemotactic factors present in plasma. Hence, natural products that effectively prohibit superoxide-dependent chemotactic factor, serve as promising anti-inflammatory agents [39]. Thus, the anti-inflammatory effect is measured through the inhibition of superoxide anion generation and elastase liberation in our ongoing study.

### 2.5. In Vitro Antioxidant Activity Using DPPH Assay

The main mechanistic basis for most in vitro antioxidant assays takes place by either HAT (hydrogen atom transfer) or ET (election transfer) reactions. DPPH is monitored by determining the color change of the DPPH radical. The DPPH assay relied upon election transfer reaction, wherein DPPH itself reacts as a radical and a probe. The presence of antioxidants in any sample lightens the color of DPPH by donating an electron/hydrogen atom to the unpaired electron of DPPH that turns the purple color of DPPH colorless, which is measured using UV–VIS spectrophotometer at a wavelength of 517 nm [40]. The DPPH assay showed that *C. aethiopica* leaf total methanol extract showed substantial antioxidant potential with an IC_50_ equals of 0.56 mg/mL that may be attributed to the richness of the extract with phytoconstituents belonging to different classes represented mainly by flavonoids and phenolic acid as revealed from the LC/MS analysis.

### 2.6. In Vitro Anti-Hyperglyceamic Activity

The anti-hyperglycemic activity of the *C. aethiopica* leaf total methanol extract (CAL) was evaluated in vitro using 3T3-L1 adipocytes using two concentrations which are 30 and 50 μg/mL that were further compared with insulin and pioglitazone, positive controls, using the same concentrations. Results displayed in Table 5 clarified that the concentration of glucose was decreased from 23.9 and 24.1 mmol/L to 22.8 and 22.6 mmol/L after treatment with 30 and 50 μg/mL of CAL, respectively. This showed a moderate reduction in the culture medium glucose concentration estimated by 4.6 and 6.2% as compared to untreated control group. CAL approaches in this regard insulin and pioglitazone that showed 6.3 and 4.37% reduction at 30 μg/mL and 10.5% reduction at 50 μg/mL as compared to untreated control group.

### 2.7. In Vivo Assessment of the Antihyperglycemic Activity

**Figure 4:**
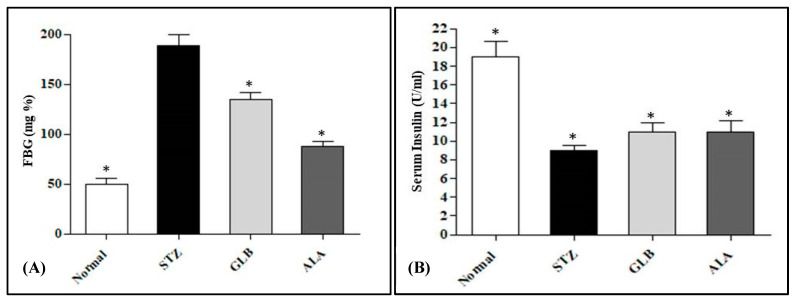
Effect of intraperitoneal injection of 20 mg/kg/day of *C. aethiopica* leaf total methanol extract (CAL) and 600 μg/kg/day glibenclamide (GLB) on FBG (**A**) and serum insulin (**B**) levels in STZ-induced diabetic rats. Data are measured in triplicates (n = 3) and presented as means ± S.E.M. * Significantly different from the STZ-treated group at *p* < 0.05; FBG was assessed using a glucose oxidase kit; serum insulin was determined by an immunoassay kit.

The antihyperglycemic effect of *C. aethiopica* leaf total methanol extract (CAL) was evaluated in STZ-induced diabetic rats. The injection of STZ into animals triggered a considerable increase in FBG level by 278% when compared to the normal control group (Figure 4). This change was concomitant with a significant reduction in the serum insulin level by 52.63%, with regard to the normal group. Meanwhile when GLB was administered to STZ-diabetic rats it caused a notable decrease in FBG level by 28.57% accompanied by a marked increase in serum insulin level by 22.22% in comparison with STZ-treated rats. However, the administration of the CAL to STZ-treated rats elicited a significant reduction in FBG level by 53.44%, as compared with the STZ-treated rats with concomitant elevation in serum insulin estimated by 22.22%. The results of CAL suggested a potent antihyperglycemic effect approaching that of GLB. The effectiveness of CAL as a promising antihyperglycemic is attributed to its richness by flavonoids and phenolic acids. These secondary metabolites were previously showed to possess antihyperglycemic activity via acting as potent *α*-glucosidase inhibitors [41,42]. It is noteworthy to mention that myricetin derivatives exhibited considerable *α*-amylase as well as *α*-glucosidase inhibitory activity. They also displayed an insulin-like action via stimulating glucose and lipid uptake as well as enhancing adiponectin production by stimulating the insulin signaling pathway in a manner similar to insulin. Myricetin derivatives trigger Akt1, protein kinase B, peroxisome proliferator-activated receptor gamma, and Slc2a4, glucose transporter, genes up-regulation resulting in antihyperglycemic activity [43]. Moreover, quinic acid and its derivatives effectively stimulate Ca^2+^-dependent mitochondrial function and increase insulin production from *β*-cells of the pancreas [44]. The plant product quinic acid activates Ca^2+^-dependent mitochondrial function and promotes insulin secretion from pancreatic beta cells. The plant product quinic acid activates Ca^2+^-dependent mitochondrial function and promotes insulin secretion from pancreatic beta cells. The plant product quinic acid activates Ca^2+^-dependent mitochondrial function and promotes insulin secretion from pancreatic beta cell [44].

### 2.8. Molecular Modeling

**Figure 5:**
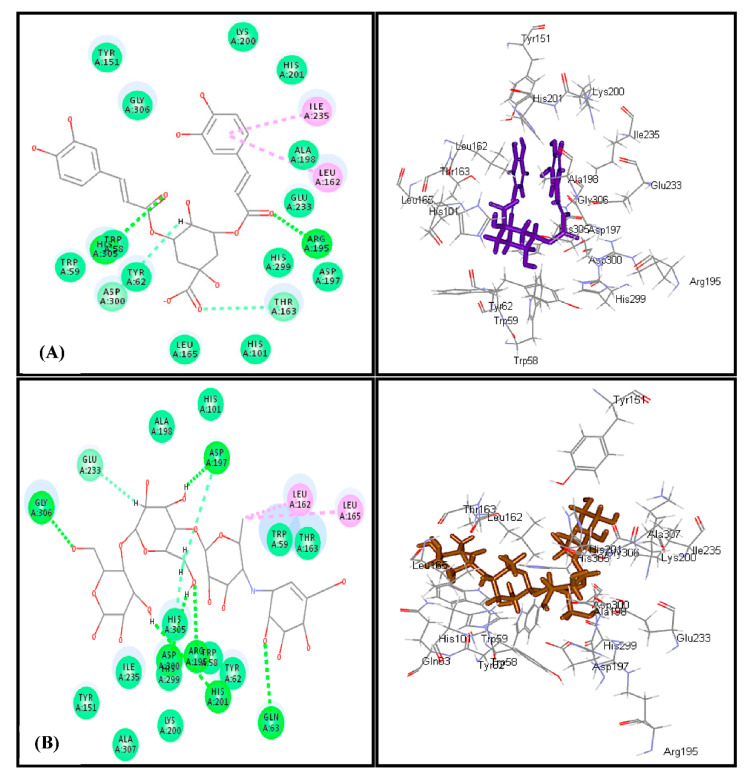
2D and 3Dbinding modes of dicaffeoylquinic acid (**A**) and acarbose (**B**) within human amylase (HA) active centers employing C-docker protocol.

**Figure 6:**
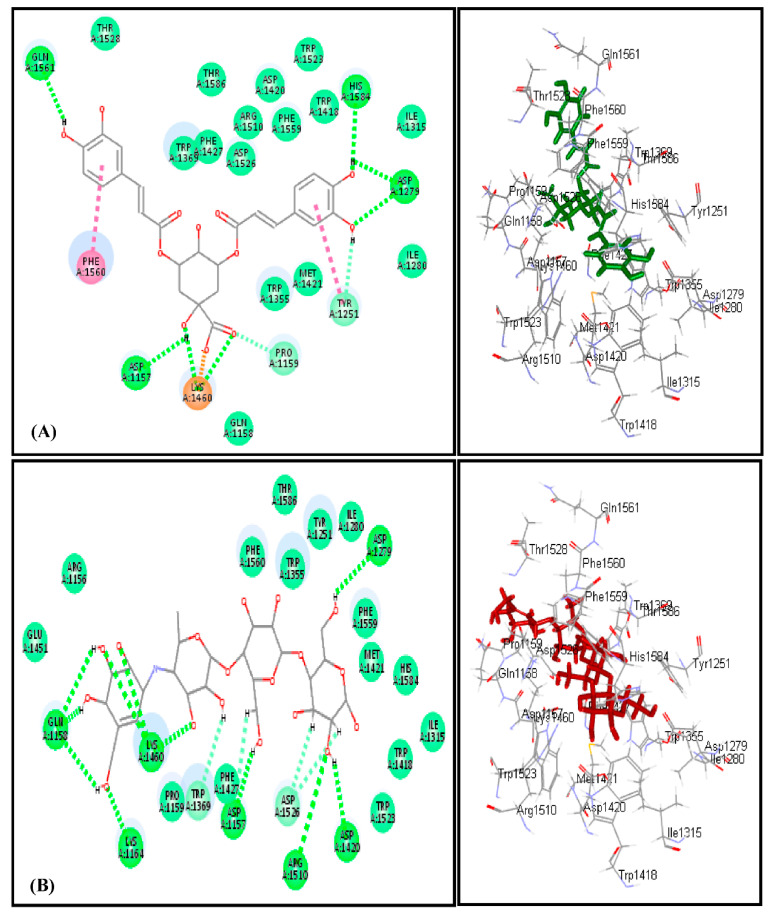
2D and 3D binding modes of dicaffeoylquinic acid (**A**) and acarbose (**B**) within human glucosidase (HG) active centers employing C-docker protocol.

Docking of most of the major compounds identified from *C. aethiopica* leaf total methanol extract inside the active centers of human *α*-amylase (HA) and human *α*-glucosidase (HG) was performed to further ascertain the results of the in vitro and in vivo study and provides an outline for the probable mechanism of action. Most of the docked compounds showed certain fitting within the active sites of both examined enzymes. Dicaffeoylquinic acid revealed the highest fitting with free binding energies (∆G) equals to −47.24 and −60.50 Kcal/mol for human *α*-amylase (HG) and human *α*-glucosidase (HG), respectively (Table 6). The highest fitting of dicaffeoylquinic acid at human *α*-amylase (HA) is attributed to the formation of two conventional H-bonds with His 305 and Arg 195, two π-alkyl interactions with Ile 235 and Leu 162, two C-H bonds with Asp 300 and Thr 163 in addition to the formation of many Van der Waals interactions. Regarding acarbose, it forms six conventional H- bonds with Gly 306, Asp197, Gln 63, His 291, Arg 195 and Asp 300, two alkyl interactions with Leu 162 and Leu 165, two C-H bonds with Glu 233 and His 305 in addition to Van der Waals interactions with many amino acid residues at the active site (Figure 5). The firm binding of dicaffeoylquinic acid at the active site of human *α*-glucosidase (HG) can be interpreted by the formation of multiple tight bonds at the active sites, namely, seven conventional H-bonds with Gln 1561, His 1584, Asp 1279, Lys 1460, and Asp 1157; two π–π interactions with Phe 1560 and Tyr 1251; and hydrophobic C-H bonds with Pro 1159 and Tyr 125 in addition to the formation of many Van der Waals interactions with most of the amino acid moieties present at the active site of the enzyme together with an attractive charged ionic bond formation between the carboxylic acid moiety of the quinic acid and Lys 1460 residue in the active site (Figure 6). These results for dicaffeoylquinic acid approached that of acarbose, the standard antihyperglycemic agent that displayed ∆G of −76.29 and −89.19 Kcal/mol for human *α*-amylase (HG) and human *α*-glucosidase (HG), respectively. Acarbose formed eleven H-bonds with Gln 1158, Lys 1460, Lys 1164, Asp 1157, Arg 1510, and Asp 1420, in addition to two C-H bonds with Trp 1369 and Asp 1526 at the human *α*-glucosidase (HG) active center.

## 3. Materials and Methods

### 3.1. Chemical Reagents and Kits

Glibenclamide (GLB), streptozotocin (STZ), [3-(4,5-dimethylthiazol-2-yl)-2,5-diphenyltetrazolium bromide] (MTT), Dulbecco’s modified Eagle’s medium-high glucose powder (DMEM), streptomycin, calcium ionophore A23187, *o*-phthalaldehyde, *p*-nitrophenyl-*N*-acetyl-d-glucosaminide (p-NAG), dexamethasone, and penicillin were bought from Sigma-Aldrich (St. Louis, MO, USA). Roche Diagnostics Germany (Mannheim, Germany) kindly supplied glucotest strips. The radioimmunoassay kit used for determination of insulin level and the glucose oxidase kit were purchased from Amersham Biosciences; (Piscataway, NJ, USA) and Siemens Healthcare Diagnostics (Deerfield, IL, USA), respectively. DNP-BSA (dinitrophenyl-conjugated bovine serum albumin) and FBS (fetal bovine serum) were obtained from Merck (Kenilworth, NJ, USA) and Hyclone (Logan, UT, USA), respectively. Other kits used for assessment of LPO (lipid peroxidation) and MDA (malondialdehyde) were obtained from Fluka (Buchs, Switzerland). Dr. Daniel H. Conrad (Virginia Commonwealth University, Richmond, VA, USA) generously supplied Mouse anti-DNP IgE antibody. All other solvents for LC/MS as well as GLC/MS analyses were of analytical grade.

### 3.2. Plant Material

*C. aethiopica* (Iridaceae) leaves were collected from El-Rai botanical garden, El Kanater El Khayreya, Qalyubiya, Egypt. The plants were kindly identified and authenticated by Mrs. Therese Labib, Consultant of Plant Taxonomy at the Ministry of Agriculture and former Director of Orman Botanical Garden, Giza, Egypt, and Dr. Mohammed El-Gebaly, Department of Botany, National Research Centre (NRC), Giza, Egypt. Voucher specimens were deposited in the herbarium of Pharmacognosy Department, Faculty of Pharmacy, Ain Shams University (voucher specimen number: PHG-P-CA-261).

### 3.3. Preparation of the Plant Extract and Its Fractions

Air-dried leaves of *C. aethiopica* L. (100 g) were powdered and percolated in 80% methanol (1 L × 3) followed by its filtration. The obtained filtrate was concomitantly evaporated under reduced pressure and temperature (45 °C) until dryness and lyophilized to give 25.50 g of the total methanol extract (CAL). The solid residue (20 g) was dissolved in water: methanol (1: 4) and then partitioned successively with *n*-hexane, dichloromethane, ethyl acetate, and *n*-butanol to give CAL-A (2.67 g), CAL-B (3.74 g), CAL-C (2.34 g), and CAL-D (1.16 g), respectively. The remaining aqueous fraction CAL-E was 9.58 g.

### 3.4. Gas–Liquid Chromatography Coupled with Mass Spectroscopy (GLC-MS) Analysis

GLC-MS analysis was achieved on Shimadzu GCMS-QP 2010 (Shimadzu Corporation, Koyoto, Japan) equipped with Rtx-5MS (30 m × 0.25 mm i.d. × 0.25 µm film thickness) capillary column (Restek, PA, USA) and coupled to a Shimadzu mass spectrometer. Initial column temperature was set at 50 °C for 3 min; 50–300 °C at a rate of 5 °C/min and then 10 min isothermal at 300 °C. The injector temperature was 280 °C. The carrier gas (helium) flow rate was set at 1.37 mL/min. The interface and ion source temperatures were adjusted to 280 and 220 °C, respectively. Diluted samples (1% *v*/*v*) were injected in split mode with split ratio 15:1. The injection volume was 1 μL. Mass spectra were recorded in EI mode of 70 eV, scanning from *m/z* 35 to 500.

#### Compound Identification

The tentative identification of the compounds found by GLC-MS was performed based on their retention indices relative to a homologous series of *n*-alkanes (C_8_–C_28_) injected under the same conditions and comparing their mass spectra with those in the National Institute of Standards and Technology (NIST) and Wiley library database in addition to literature [45,46,47,48,49,50,51,52].

### 3.5. HPLC-PDA-ESI-MS/MS Analysis

HPLC-PDA-ESI-MS/MS analysis was carried out on a Finnigan LCQ-Duo ion trap mass spectrometer (Thermo Quest, San Jose, CA, USA) with an ESI source coupled to a Finnigan Surveyor HPLC system (Accela autosampler, MS pump plus, and PDA detector plus) (Thermo, San Jose, CA, USA) with an EC 150/3 Nucleodur 100-3 C18ec column (Macherey-Nagel, Düren, Germany). A gradient of water and acetonitrile (ACN) (with 0.1% formic acid for ESI^+^ mode and without formic acid for ESI^−^ mode) was applied from 2% to 100% ACN in 60 min at 30 °C at a flow rate of 0.5 mL/min. The injection volume was 20 µL. All samples were measured in the positive and negative ion mode. The MS was operated at +10 V capillary voltage for ESI^+^ and −10 V for ESI^−^, 240 °C source temperature, and high purity nitrogen as a sheath and auxiliary gas at a flow rate of 80 and 40 (arbitrary units), respectively. The ions were detected in a mass range of 50–2000 *m/z*. A collision energy of 35 eV was used in MS/MS fragmentation. Data acquisitions and analyses were executed by Xcalibur^TM^ 2.0.7 software (Thermo Scientific, Karlsruhe, Germany). Tentative metabolite assignments were achieved by comparison of mass and UV spectral data of the detected compounds in both negative and positive ionization modes with reported data alongside online public databases where references are added Table 2.

### 3.6. In Vitro Biological Evaluation

#### 3.6.1. In Vitro Assessment of the Anti-Allergic Activity

##### Cell Culture and Cell Viability Assay

The mucosal mast cell-derived rat basophilic leukemia (RBL-2H3) cell line was obtained from the American Type Culture Collection. The growth of RBL-2H3 was carried out in a DMEM medium containing 10% FBS in addition to 100 U/mL penicillin and 100 μg/mL streptomycin. The culturing of cells was done in 10 cm cell culture dishes (Cellstar) using a humidified chamber containing 5% CO_2_ in the air and were kept at 37 °C. A methyl thiazole tetrazolium (MTT) assay was performed to assess the probable toxic effects of the tested samples concerning RBL-2H3 cells [53] as previously described [54,55,56,57].

##### Degranulation Assays in Mast Cells

The extent of A23187-induced degranulation in RBL-2H3 cells was evaluated using a *β*-hexosaminidase activity assay as formerly described [54,58]. The release of *β*-hexosaminidase from activated RBL-2H3 cells induced by IgE was assessed following the method described earlier [59]. Dexamethasone (10 nM) was used as a positive control. Absorbance was determined using a microplate reader at *λ* = 405 nm. The percentage of inhibition of *β*-hexosaminidase release from RBL-2H3 cells was computed using the following equation, calculated as a percentage of the control value (untreated stimulated cells:

$$\mathrm{Inhibition}\;\left(\%\right)=\left[1-\frac{\left(\mathrm{ODsample}-\mathrm{ODspontaneous}\right)}{\left(\mathrm{ODcontrol}-\mathrm{ODspontaneous}\right)}\right]\times 100$$

#### 3.6.2. In Vitro Assessment of Anti-Inflammatory Activity

The assessment of anti-inflammatory activity was performed using human neutrophils in which blood was withdrawn from healthy volunteers (20–35 years old) following a protocol approved by the institutional review board at Chang Gung Memorial Hospital adopting a standard method as formerly described [60]. However, the inhibition of superoxide generation was assessed using the assay based on the reduction of ferricytochrome c as previously reported [60]. Meanwhile, the release of elastase from activated neutrophils was measured using the elastase substrate, *N*-methoxysuccinyl-Ala-Pro-Val-*p*-nitroanilide, following the previously described procedure [60].

#### 3.6.3. In Vitro Antioxidant Activity Evaluation Using DPPH Assay

The ability of CAL to donate hydrogen atom or an electron was determined via bleaching the purple color of DPPH^•^ methanol solution 0.2, 0.4, 0.6, and 1.0 mg/mL that was performed in triplicate. Briefly, 50 μL of each concentration of the tested sample was added to 5 mL of 0.004% methanol solution of DPPH. After 30 min incubation at room temperature, the absorbance was measured versus a blank at *λ* = 517 nm using a Shimadzu UV-1601 spectrophotometer (Kyoto, Japan). The percentage of inhibition of DPPH^•^ was calculated using the following equation: Free radical scavenging activity = [Ac − As/Ac] × 100, where Ac: absorbance of control and As: absorbance of sample. The IC_50_ of ascorbic acid, positive control, was 1.6 μg/mL [61].

#### 3.6.4. In Vitro Anti-Hyperglycemic Evaluation Using 3T3-L1 Adipocyte Culture

Stock solutions of the tested sample at 30 and 50 μg/mL in DMSO were used in which DMSO concentration does not exceed 0.1% in the medium of working solutions. However, the stock solution of insulin was prepared by dissolving 10^2^ M of insulin in 0.01 M of acetic acid; pH 3.0 [62]. 5 × 10^5^ cells/mL of mouse pre-adipocytes 3T3-L1 cells obtained from the American Type Culture Collection (ATCC) were used and cultured in DMEM medium supplemented with 5.56 mmol/L D-glucose, 10% FBS, and 1% PS (penicillin–streptomycin) solutions. The cells were kept at 37 °C in a humidified condition composing of 95% air and 5% CO_2_. After achieving 100% confluence, the cells were treated with 25 mmol/L D-glucose, 0.32 μM insulin, 0.5 mM 3-isobutyl-1-methylxanthine, and 1 μM dexamethasone for 48 h to improve their differentiation. Cells treated with CAL as well as control untreated cells were kept in DMEM media with 25 mmol/L D-glucose for 24 h. Pioglitazone and insulin were employed as a positive control meanwhile the concentrations of glucose in the medium were employed as a tool to assess anti-hyperglycemic effectiveness of the tested sample [62].

### 3.7. In Vivo Biological Evaluation

#### 3.7.1. Animals and Animal Treatment

Rats (Wister male) of 150–250 g, were provided by King Fahd Medical Research Center, King Abdulaziz University, Jeddah, Saudi Arabia, animal facility. The study protocol received approval from the local ethics committee for animal care, Taibah University, Saudi Arabia (Approval number TUCDREC/20160131). The animals were kept under standard conditions of temperature, relative humidity, as well as day and light cycle. Animals were allowed free access to standard laboratory food as previously reported [62] and as indicated by the WHO (World Health Organization) [62].

#### 3.7.2. Diabetes Induction in Rats

The induction of diabetes in rats was achieved by the intraperitoneal administration of streptozotocin (mg/kg) as a single dose as previously described [63]. Enzymatic strips were used to examine the existence of glucose in the urine on the third day. Glibenclamide was utilized as a positive control.

#### 3.7.3. Experimental Protocol

The animals were randomly distributed into four groups, 8 animals per group. The experimental protocol was done following what was previously reported in which groups 1, 2, and 3 represented the normal, streptozotocin (STZ), and glibenclamide (GLB) administered groups, respectively [62]. The fourth group orally received 20 mg/kg body weight of the leaf methanol extract of *C. aethiopica* L. (CAL) for ten successive days. Scarification was done on the 11th day of treatment by cervical dislocation after light ether anesthesia. The separated serum was subjected to centrifugation and used for the determination of glucose and insulin levels.

#### 3.7.4. Assessment of the Biochemical Parameters

Serum glucose and insulin were assessed using the glucose oxidase assay and Amersham insulin immune reactive kit, respectively [62,64], as listed by the manufacturer [65].

### 3.8. Statistical Analysis

Statistical analysis was done using one-way ANOVA followed by Dunnet’s test which was done using Sigma Plot (Systat software, San Jose, CA, USA) in case of anti-allergic evaluation; student’s *t*-test (SigmaPlot) for anti-inflammatory assay; however, for the antihyperglycemic determination, ANOVA was followed by Tukey–Kramer multiple comparison test (*p* < 0.05). Results were given as means ± S.E.M. Graphs were plotted using GraphPad Prism 6 software (GraphPad Inc., La Jolla, CA, USA).

### 3.9. Molecular Modeling

Molecular docking of the identified compounds from the total methanol leaf extract (CAL) was performed using Discovery Studio 2.5 (Accelrys Inc., San Diego, CA, USA) implementing C-docker protocol on human *α*-amylase (HA) (PDB ID 1B2Y) and human*α*-glucosidase (HG) (PDB ID 3TOP), retrieved from the protein data bank (www.pdb.org, accessed on 28 May 2021) using both pH based and rule-based methods. This was done following the previously reported method [3,35,66].

## 4. Conclusions

This study comprehensively studies the phytoconstituents as well as the different biological activities of the *C. aethiopica* for the first time. GC analyses revealed that palmitic acid and methyl hexadecanoic acid ester are the predominant compounds in *C. aethiopica n*-hexane fraction of the leaf extract. Meanwhile, myricetin-3-*O*-rhamnoside was the major compound in *C. aethiopica* total methanol extract as displayed in HPLC-PDA-ESI-MS/MS analysis. *C. aethiopica n*-hexane exhibited the highest anti-allergic and anti-inflammatory potential whereas *C. aethiopica* total methanol extract showed a potent antihyperglycemic activity in streptozotocin-induced diabetes in an animal model that was further confirmed using in silico studies. *C. aethiopica* could serve as an effective antioxidant natural remedy combating inflammation, allergy, and hyperglycemia that will be welcomed by a large category of patients worldwide. Further studies are recommended for the isolation and structural elucidation of the compounds existing in the extract with subsequent assessment of their biological activities.

## Figures and Tables

**Table 1:** Chemical profile of *C. aethiopica* L. *n*-hexane fraction (CAL-A) by GLC-MS.

No.	Rt	Compound Name	RI_Exp._ *^a^*	RI_Lit_ *^b^*	Content%	Molecular Formula	Identification *^c^*
1.	31.44	Methyl pentadecanoic acid ester	1806	1812	0.15	C_16_H_32_O_2_	MS, RI
2.	31.68	Neophytadiene	1818	1817	0.50	C_20_H_38_	MS, RI
3.	31.85	Hexahydrofarnesyl acetone	1826	1825	5.30	C_18_H_36_O	MS, RI
4.	33.47	7-Hexadecenoic acidmethyl ester (Z)	1903	1900	0.68	C_17_H_34_O_2_	MS, RI
5.	33.54	Hexadecanoic acid methyl ester	1907	1907	11.91	C_17_H_34_O_2_	MS, RI
6.	34.45	Palmitic acid	1950	1950	16.08	C_16_H_32_O_2_	MS, RI
7.	34.99	Heptadecanoic acid methyl ester	1976	1978	0.14	C_18_H_36_O_2_	MS, RI
8.	35.55	15-Methyl hexadecanoic acid methyl ester	2003	1996	0.52	C_18_H_36_O_2_	MS, RI
9.	36.77	16-Methyl heptadecanoic acid methyl ester	2070	2077	0.25	C_19_H_38_O_2_	MS, RI
10.	36.90	Linoleic acid methyl ester	2077	2075	2.42	C_19_H_34_O_2_	MS, RI
11.	37.03	Oleic acidmethyl ester	2085	2085	4.10	C_19_H_36_O_2_	MS, RI
12.	37.25	Phytol	2096	2096	1.50	C_20_H_40_O	MS, RI
13.	37.48	Methyl stearate	2109	2109	2.08	C_19_H_38_O_2_	MS, RI
14.	37.89	Linolenic acid	2131	2134	3.74	C_18_H_30_O_2_	MS, RI
15.	38.24	Stearic acid	2150	2155	0.83	C_18_H_36_O_2_	MS, RI
16.	41.08	Eicosanoic acid methyl ester	2306	2307	0.57	C_21_H_42_O_2_	MS, RI
17.	41.62	4,8,12,16-Tetramethylheptadecan-4-olide	2336	2364	1.99	C_21_H_40_O_2_	MS
18.	42.84	2,2′-Methylene-bis-(6-tert butyl-4-methylphenol)	2403	2398	0.13	C_23_H_32_O_2_	MS, RI
19.	43.86	*n*-Pentacosane	2469	2500	1.46	C_25_H_52_	MS
20.	44.22	Palmitic acid *β*-monoglyceride	2492	2498	0.16	C_19_H_38_O_4_	MS, RI
21.	44.41	Docosanoic acid methyl ester Behenic acid methyl ester	2504	2492	0.40	C_23H46_O_2_	MS, RI
22.	45.44	11-Methylpentacosane	2570	2565	1.99	C_26_H_54_	MS, RI
23.	45.97	Tricosanoic acid methyl ester	2605	2615	0.05	C_24_H_48_O_2_	MS, RI
24.	46.95	2-Methylhexacosane	2668	2663	2.41	C_27_H_56_	MS, RI
25.	47.49	Tetracosanoic acid methyl ester	2702	2714	0.14	C_25_H_50_O_2_	MS, RI
26.	48.42	2-Methylheptacosane	2762	2762	2.47	C_28_H_58_	MS
27.	49.43	*α*-Tocospiro A	2827	2860	4.13	C_29_H_50_O_4_	MS
28.	49.74	*α*-Tocospiro B	2847	2881	4.62	C_29_H_50_O_4_	MS
29.	49.83	2-Methyl octacosane,	2852	2857	3.57	C_29_H_60_	MS, RI
30.	50.20	Cholesta-2,4-diene	2876	2872	0.09	C_27_H_44_	MS, RI
31.	51.19	15-Methylnonacosane	2940	2935	2.05	C_30_H_62_	MS, RI
32.	52.51	*n*-Triacontane	3025	3003	2.02	C_30_H_62_	MS, RI
33.	52.94	*β*-Sitosterol propionate	3053	-	1.77	C_32_H_54_O_2_	MS
34.	53.31	*α*-Tocopherol	3076	3112	1.76	C_29_H_50_O_2_	MS
35.	53.84	Hentriacontane	3110	3103	1.33	C_31_H_64_	MS, RI
36.	55.31	Dotriacontane	3205	3202	0.64	C_32_H_66_	MS, RI
37.	56.36	Chondrillasterol	3272	3295	1.54	C_29_H_48_O	MS, RI
38.	57.16	*β*-Amyrin	3323	3337	0.42	C_30_H_50_O	MS, RI
39.	57.43	*γ*-Sitosterol	3341	3351	0.17	C_29_H_50_O	MS, RI
40.	58.06	*α*-Amyrin	3381	3376	1.53	C_30_H_50_O	MS, RI
41.	58.23	Stigmasta-3,5-dien-7-one	3392	-	0.29	C_29_H_46_O	MS
42.	59.07	*β*-Amyrin acetate	3446	3438	0.76	C_32_H_52_O_2_	MS, RI
43.	60.05	Lupeol acetate	3509	3525	0.64	C_32_H_52_O_2_	MS, RI
44.	60.42	Hexadecanoic acid, 3,7,11,15-tetramethyl-2-hexadecenyl ester	3533	3568	2.32	C_36_H_70_O_2_	MS, RI
		Total identified (%)			91.62		

*^a^* Retention index determined experimentally on RTX-5MS column relative to *n*-alkane series (C8–C28), *^b^* Published retention indices, *^c^* Identification was based on comparison of mass spectral data (MS) and retention indices (RI) with those of NIST Mass Spectral Library (2017), Wiley Registry of Mass Spectral Data 8th edition and literature.

**Table 2:** Metabolite profiling of *C. aethiopica* leaf total methanol extract via HPLC-PDA-ESI-MS/MS in the positive and negative ion mode.

Peak No.	*t_R_* (min)	Name	UV λ_max_ (nm)	[M − H]^+^ (*m/z*)	[M − H]^−^ (*m/z*)	MS^2^ (*m/z*)	Molecular Formula	References
1.	2.59	Quinic acid	274		191	173, 127, 85	C_7_H_12_O_6_	[16]
2.	5.09	Phenyl alanine	252, 275	166	164	147, 119	C_9_H_11_NO_2_	[17]
3.	5.75	Protocatechuic acid	254, 286		153	109	C_7_H_6_O_4_	[18]
4.	7.21	Dicaffeoyl quinic acid	269, 310		515	353, 341, 323, 191, 179	C_25_H_24_O_12_	[19]
5.	7.54	Dicaffeoyl quinic acid isomer	282, 310		515	353, 341, 323, 191, 179	C_25_H_24_O_12_	[19]
6.	7.65	Hydroxy-methoxy acetophenone-*O*-hexoside	283		327	295, 283, 179, 165,147, 119	C_15_H_20_O_8_	[20] [21]
7.	8.07	Caffeoylquinic acid	289, 322		353	191, 179, 135	C_16_H_18_O_9_	[22]
8.	9.24	Caffeoylquinic acid isomer	288, 315		353	191, 179, 135	C_16_H_18_O_9_	[22]
9.	9.88	Myricetin-*O*-coumaroyl- hexoside	270, 316		625	479, 478, 463, 317, 271, 179	C_30_H_26_O_15_	[23]
10.	10.34	Iristectorin A/B (3′,5,7-Trihydroxy-4′,6-dimethoxy-isoflavone-7-*O*-hexoside)	265		491	473, 447, 401, 371, 343, 329, 311, 283, 241, 191, 146,	C_23_H_24_O_12_	[24] [25]
11.	11.12	Myricetin-*O*-coumaroyl-hexoside	263, 311		625	479, 478, 463, 317, 287, 271	C_30_H_26_O_15_	[23]
12.	11.75	Tricin-*O*-rhamnoside (Trihydroxy-dimethoxy-flavone-*O*-rhamnoside)	264, 310	477	475	329, 328, 315, 299	C_23_H_24_O_11_	[26]
13.	13.33	Trihydroxy-dimethoxy-flavone-*O*-rhamnoside	265, 310	477	475	457, 447, 431, 329, 328, 301, 175, 145	C_23_H_24_O_11_	[26]
14.	14.21	Hexahydroxy-isoflavone-*C*-hexoside (Irigenol-*C*-hexoside)	264, 322		479	461, 433, 389, 359, 317, 316, 271, 151	C_21_H_20_O_13_	[27]
15.	14.92	Myricetin-3-*O*-rhamnoside	255, 346	465	463	317, 316, 287, 271, 262, 179, 151	C_21_H_20_O_12_	[23]
16.	15.36	Myricetin	255, 350	319		319, 301, 273, 263, 165, 179, 153, 109	C_15_H_10_O_8_	[28]
17.	15.87	Tetrahydroxy dimethoxy isoflavone-*O*-hexoside (Hydroxy-iristectrigenin-*O*-hexoside)	234, 270, 311	509	507	489, 476, 475, 463, 345, 329, 301, 273, 191, 175	C_23_H_24_O_13_	[29]
18.	17.19	6-Hydroxyluteolin 7-*O*-rhamnoside	316		447	301	C_21_H_20_O_11_	[30]
19.	17.46	6-Hydroxy luteolin	234, 315	303		303, 285, 257, 165, 153, 137	C_15_H_10_O_7_	[30]
20.	17.75	Myricetin-*p*-coumaroyl-rhamnosyl-hexoside	316	773	771	625, 463, 317	C_36_H_36_O_19_	[31]
21.	23.20	5,4′-Dihydroxy-3′-methoxy-6,7-methylenedioxy isoflavone (Iriflogenin)	276		327	309, 291, 283, 180, 165, 137	C_17_H_12_O_7_	[32]
22.	23.73	Dihydroxy-methoxy-6,7-methylenedioxy isoflavone	276		327	309, 291, 283, 229, 179, 165	C_17_H_12_O_7_	[32]
23.	29.14	Dihydroxy-23-oxo-12-oleanen-28-oic acid-*O*-pentosyl dihexoside	275		941	779, 617, 485	C_48_H_77_O_18_	[33]
24.	29.41	Dihydroxy-octadecadienoic acid (Dihydroxy-linoleic acid)	274		311	293, 267, 171, 153	C_18_H_32_O_4_	[15]
25.	35.93	Hydroxy octadecatrienoic acid (Hydroxy-linolenic acid)	276		293	275, 249, 211, 183, 171, 121	C_18_H_30_O_3_	[15]
26.	38.45	Hydroxy octadecadienoic acid (Hydroxy-linoleic acid)	277		295	277, 251, 211, 195, 183, 171	C_18_H_32_O_3_	[15]
27.	41.33	Hydroxy octadecenoic acid (Hydroxy-oleic acid)	276		297	279, 253, 171	C_18_H_34_O_3_	[15]
28.	48.29	Hydroxyhexadecanoic acid (Hydroxy-palmitic acid)	Nd		271	253, 225, 210	C_16_H_32_O_3_	[18]
29.	54.51	Hydroxyoctadecanoic acid (Hydroxy-stearic acid)	Nd		299	253	C_18_H_36_O_3_	[18]

**Table 3:** Anti-allergic effects of *C. aethiopica* leaf total methanol extract and its fractions at different concentrations using degranulation assay in RBL-2H3 cell line.

Sample	Inhibition % of A23187-Induced *β*-Hexosaminidase Release ^a^				Inhibition % of Antigen-Induced *β*-Hexosaminidase Release ^a^			
	1 μg/mL	10 μg/mL	100 μg/mL	200 μg/mL	1 μg/mL	10 μg/mL	100 μg/mL	200 μg/mL
CAL	NS ^c^	TOX ^d^	TOX ^d^	TOX ^d^	NS ^c^	TOX ^d^	TOX ^d^	TOX ^d^
CAL-A	NS ^b^	8.0 ± 4.9	41.3 ± 5.4 ***	72.7 ± 2.2 ***^e^	NS ^b^	4.0 ± 1.7	12.0 ± 5.2	48.7 ± 8.6 ***
CAL-B	NS ^c^	TOX ^d^	TOX ^d^	TOX ^d^	NS ^c^	TOX ^d^	TOX ^d^	TOX ^d^
CAL-C	NS ^c^	TOX ^d^	TOX ^d^	TOX ^d^	NS ^c^	TOX ^d^	TOX ^d^	TOX ^d^
CAL-D	NS ^c^	TOX ^d^	TOX ^d^	TOX ^d^	NS ^c^	TOX ^d^	TOX ^d^	TOX ^d^
CAL-E	NS ^c^	TOX ^d^	TOX ^d^	TOX ^d^	NS ^c^	TOX ^d^	TOX ^d^	TOX ^d^

CAL: *C. aethiopica* leaf total methanol extract; CAL-A: *C. aethiopica n*-hexane fraction; CAL-B: *C. aethiopica* dichloromethane fraction; CAL-C: *C. aethiopica* ethyl acetate fraction; CAL-D: *C. aethiopica n*-butanol fraction; CAL-E: *C. aethiopica* remaining aqueous fraction. ^a^ Results are presented as mean ± S.E.M. value (n = 3). *** *p* < 0.001 compared with the control value (A23187 or antigen only). Dexamethasone, a positive control, inhibited 80.7 ± 3.8% (A23187-induced) and 79.7 ± 2.5% (antigen-induced) degranulation at a concentration of 10 nM. ^b^ NS: Not significant inhibition (degranulation more than 85 % of control) (n = 1). ^c^ NS: Not significant inhibition (degranulation more than 85 % of control) (n = 2). ^d^ TOX: toxic (viability less than 80% of control). ^e^ CAL-A inhibited A23187-induced *β*-hexosaminidase release with IC_50_ 127.7 μg/mL.

**Table 4:** Inhibitory effects of *C. aethiopica* leaf total methanol extract and its fractions on superoxide anion generation and elastase release in FMLF/CB-induced human neutrophils.

Sample	Superoxide Anion		Elastase Release	
	IC_50_ (μg/mL) ^a^	Inhibition%	IC_50_ (μg/mL) ^a^	Inhibition%
**CAL**	>10	36.45 ± 2.58 ***	>10	15.27 ± 2.13 **
**CAL-A**	**5.32 ± 0.46**	68.68 ± 3.87 ***	**5.80 ± 0.34**	65.18 ± 6.81 ***
**CAL-B**	**7.04 ± 1.28**	58.31 ± 3.76 ***	**5.73 ± 0.64**	68.08 ± 7.25 ***
**CAL-C**	>10	43.84 ± 1.94 ***	>10	24.51 ± 4.05 **
**CAL-D**	>10	22.47 ± 5.53 *	^b^	^b^
**CAL-E**	>10	26.49 ± 2.73 ***	>10	5.30 ± 2.67

CAL: *C. aethiopica* leaf total methanol extract; CAL-A: *C. aethiopica n*-hexane fraction; CAL-B: *C aethiopica* dichloromethane fraction; CAL-C: *C. aethiopica* ethyl acetate fraction; CAL-D: *C. aethiopica n*-butanol fraction; CAL-E: *C. aethiopica* remaining aqueous fraction. ^a^ Percentage of inhibition at 10 μg/mL concentration. Results are presented as mean ± S.E.M. (n = 3). * *p* < 0.05, ** *p* < 0.01, *** *p* < 0.001 compared with the control value (fMLF/CB). ^b^ Sample CAL-D had promoting effects (23.86 ± 3.78% in the presence of CB) on elastase release in human neutrophils.

**Table 5:** Effect of *C. aethiopica* leaf total methanol extract (CAL) at concentrations of 30 and 50 μg/mL on glucose consumption in media of 3T3-L1 adipocyte cultures.

	Glucose Concentration in mmol/L	
	30 μg/mL	50 μg/mL
**Pioglitazone**	22.9 ± 0.7	21.10 ± 0.6 *
**Insulin**	22.4 ± 0.4 *	21.56 ± 0.7 *
**CAL**	22.8 ± 0.9	22.6 ± 0.1 *
**Control**	23.9 ± 0.7	24.1 ± 0.6
**Medium**	25.3 ± 0.6	25.0 ± 0.6

Data are measured in triplicates (n = 3) and presented as means ± SEM. * Significantly different from the untreated control group at *p* < 0.05.

**Table 6:** Free binding energies (∆G) of the major identified compounds with human *α*-amylase (HA) and human *α*-glucosidase (HG) enzymes active centers using molecular docking with pH- and rule-based methods and expressed in kcal/mol.

Compound	Human *α*-Amylase (HA)		Human *α*-Glucosidase (HG)	
	pH-Based	Rule-Based	pH-Based	Rule-Based
Quinic acid	−15.49	−15.49	−15.23	−15.23
Phenyl alanine	−32.81	−32.81	−28.53	−28.53
Protocatecheuic acid	−31.44	−31.40	−31.84	−31.80
Dicaffeoylquinic acid	−47.24	−47.24	−60.50	−60.50
Caffeoyl quinic acid	−38.43	−38.43	−37.68	−37.68
Iristectorin	−4.17	−4.17	−14.84	−14.84
Myricetin-3-*O*-rhamnoside	−26.44	−21.14	−27.30	−22.92
Myricetin	−44.05	−41.05	−45.83	−41.82
6-Hydroxyluteolin 7-*O*-rhamnoside	−39.66	−20.66	−45.68	−23.64
6-Hydroxyluteolin	−33.45	−21.66	−36.10	−23.82
Iriflogenin	−11.57	−11.57	−13.41	−13.41
Dihydroxy-linoleic acid	−23.28	−23.28	−29.60	−29.60
Hydroxy-linolenic acid	−3.89	−3.69	0.17	0.12
Hydroxy-oleic acid	−32.61	−32.61	−35.86	−35.86
Hydroxy-stearic acid	−44.62	−44.62	−52.17	−52.17
Hydroxy-palmitic acid	−44.62	−44.62	−48.43	−48.43
Acarbose	−76.29	−76.29	−89.19	−89.19

## Data Availability

Data are available upon request from the first author.
